# An app with brief behavioural support to promote physical activity after a cancer diagnosis (APPROACH): study protocol for a pilot randomised controlled trial

**DOI:** 10.1186/s40814-022-01028-w

**Published:** 2022-03-29

**Authors:** P. Lally, N. Miller, A. Roberts, R. J. Beeken, D. M. Greenfield, H. W. W. Potts, N. Counsell, N. Latimer, C. Thomas, L. Smith, J. Gath, F. Kennedy, C. Martin, L. Wyld, A. Fisher

**Affiliations:** 1 Behavioural Science and Health, UCL, Gower Street, London, WC1E 6BT UK; 2 Leeds Institute of Health Sciences, University of Leeds, Leeds, LS2 9JT UK; 3 Sheffield Teaching Hospitals NHS FT, Weston Park Hospital, Sheffield, S10 2SJ UK; 4 Institute of Health Informatics, University College London, London, UK; 5 Cancer Research UK & UCL Cancer Trials Centre, Cancer Institute, University College London, London, UK; 6 School of Health and Related Research, University of Sheffield, Sheffield, S1 4DA UK; 7 The Centre for Health, Performance, and Wellbeing, Anglia Ruskin University, Cambridge, CB1 1PT UK; 8 Yorkshire and Humberside Consumer Research Panel, http://www.yhcrp.org.uk; 9 Department of Oncology and Metabolism, University of Sheffield, Beech Hill Road, Sheffield, S10 2RX UK

**Keywords:** Cancer, Pilot, RCT, Brisk walking, Physical activity, App, Habit

## Abstract

**Background:**

There are multiple health benefits from participating in physical activity after a cancer diagnosis, but many people living with and beyond cancer (LWBC) are not meeting physical activity guidelines. App-based interventions offer a promising platform for intervention delivery. This trial aims to pilot a theory-driven, app-based intervention that promotes brisk walking among people living with and beyond cancer. The primary aim is to investigate the feasibility and acceptability of study procedures before conducting a larger randomised controlled trial (RCT).

**Methods:**

This is an individually randomised, two-armed pilot RCT. Patients with localised or metastatic breast, prostate, or colorectal cancer, who are aged 16 years or over, will be recruited from a single hospital site in South Yorkshire in the UK. The intervention includes an app designed to encourage brisk walking (Active 10) supplemented with habit-based behavioural support in the form of two brief telephone/video calls, an information leaflet, and walking planners. The primary outcomes will be feasibility and acceptability of the study procedures. Demographic and medical characteristics will be collected at baseline, through self-report and hospital records. Secondary outcomes for the pilot (assessed at 0 and 3 months) will be accelerometer measured and self-reported physical activity, body mass index (BMI) and waist circumference, and patient-reported outcomes of quality of life, fatigue, sleep, anxiety, depression, self-efficacy, and habit strength for walking. Qualitative interviews will explore experiences of participating or reasons for declining to participate. Parameters for the intended primary outcome measure (accelerometer measured average daily minutes of brisk walking (≥ 100 steps/min)) will inform a sample size calculation for the future RCT and a preliminary economic evaluation will be conducted.

**Discussion:**

This pilot study will inform the design of a larger RCT to investigate the efficacy and cost-effectiveness of this intervention in people LWBC.

**Trial registration:**

ISRCTN registry, ISRCTN18063498. Registered 16 April 2021.

**Supplementary Information:**

The online version contains supplementary material available at 10.1186/s40814-022-01028-w.

## Background

There are approximately 2.9 million people living with and beyond cancer (LWBC) in the UK, expected to rise to 4 million by 2030 [1]. People LWBC are at risk of adverse consequences of cancer and treatments, including fatigue, pain, osteoporosis, hypertension, cardiovascular disease, secondary cancers, anxiety, fear of cancer recurrence, and depression [2–6]. As a result, people LWBC often experience poorer quality of life and reduced survival when compared to the general population [2, 3, 7]. Developing interventions that can mitigate some of the negative effects experienced by people LWBC is therefore a public health priority [8].

A large body of evidence shows that physical activity has multiple benefits following a cancer diagnosis. Observational data suggest that people LWBC who are more active have reduced risk of cancer-specific and all-cause mortality (in the region of 25–41%), reduced risk of cancer recurrence, and experience less fatigue, pain, anxiety, depression, sleep disturbance, and better quality of life [9–11]. A recent review, including 679 exercise trials in people LWBC demonstrated that exercise training is safe across the cancer continuum and has beneficial outcomes on both physical and psychological functioning [12]. The evidence for the benefits of physical activity is particularly strong for breast, prostate, and colorectal cancer, three of the most commonly diagnosed cancers worldwide [12, 13]. Given the benefits, the World Cancer Research Fund recommends that people LWBC should aim for ≥ 150 min of at least moderate-intensity physical activity per week [14]. However, it is estimated that less than 30% of people LWBC are meeting these guidelines [15].

The Independent Cancer Taskforce has recommended that everyone diagnosed with cancer in the UK should receive physical activity advice as part of their routine care [16]. However, research from our group found that people LWBC often do not receive physical activity advice from oncology professionals as part of standard care, despite a desire to receive it [17–19]. Healthcare professionals (HCPs) report multiple barriers to delivery, including lack of knowledge of guidelines, feeling that they are not the ‘right person,’ and lack of time and resources [20]. This highlights the need for interventions that are feasible for implementation into care and accessible to a large number of patients. Many trials demonstrating the benefits of physical activity interventions after cancer are supervised by trained professionals, delivered in hospital or community settings, which can lead to high associated costs and limited accessibility [21]. In addition, the COVID-19 pandemic has put pressure on cancer services and changed models of care so it is likely that at least partial remote delivery will be required [22].

Digital interventions have potential for remote delivery of interventions, and smartphone apps are well-positioned as a platform due to their popularity and capabilities. Smartphone ownership continues to increase in all age groups; in 2021, 94% of adults aged over 55 in the UK owned a mobile phone, 83% of which were smartphones [23, 24]. Smartphone apps can track physical activity, deliver ‘in-the-moment’ behaviour change support, and, once developed, can be relatively cost-effective. A meta-analysis of 15 studies conducted by our group found that digital interventions could increase moderate-to-vigorous physical activity (MVPA) participation by approximately 40 min per week in people LWBC [25]. However, only two of these interventions were delivered via apps, and most were small pilot studies and used self-reported physical activity [25]. A subsequent review indicated that smartphone interventions may increase physical activity in people LWBC and that incorporating some element of personal contact could enhance efficacy [26]. This review also highlighted the importance of assessing cost-effectiveness [26]. In order for interventions to have a positive impact on long-term health, they need to promote behaviour change that will be maintained. One route to behaviour maintenance is establishing habits; behaviours which are cued by the contexts in which they are performed, rather than intentionally selected on each occasion they are performed [27]. Habit theory provides a basis on which to provide guidance to help people develop habits [28]. An additional consideration for app-based interventions is a need for sustainability beyond the end of research funding, so utilising/adapting publicly or commercially available apps could have potential.

The design of this study was further informed by qualitative user experience research evaluating existing, publicly available physical activity apps with 31 people diagnosed with breast, prostate, and colorectal cancer. This study identified that people LWBC reported a preference for an app-based physical activity intervention that targeted walking, had elements of tailoring to/recognition of their ability and cancer side-effects, and was endorsed by oncology HCPs and professional bodies [29]. Further qualitative interviews with 19 oncology clinical nurse specialists found that they were generally positive about physical activity apps and felt walking apps would be suitable for their patients before, during, and after treatment [30]. However, they highlighted the need for demonstrated efficacy before they would be willing to recommend them as part of cancer care [30]. Therefore, the ultimate aim of this work is to test the efficacy of an app-based walking intervention, informed by habit theory, and delivered to patients with breast, prostate, and colorectal cancer during their cancer care. The aims of the pilot study are to investigate the feasibility and acceptability of the outcome measures and procedures and obtain initial estimates of the parameters for the intended future primary outcome (device-measured physical activity).

## Trial design

**Fig. 1:**
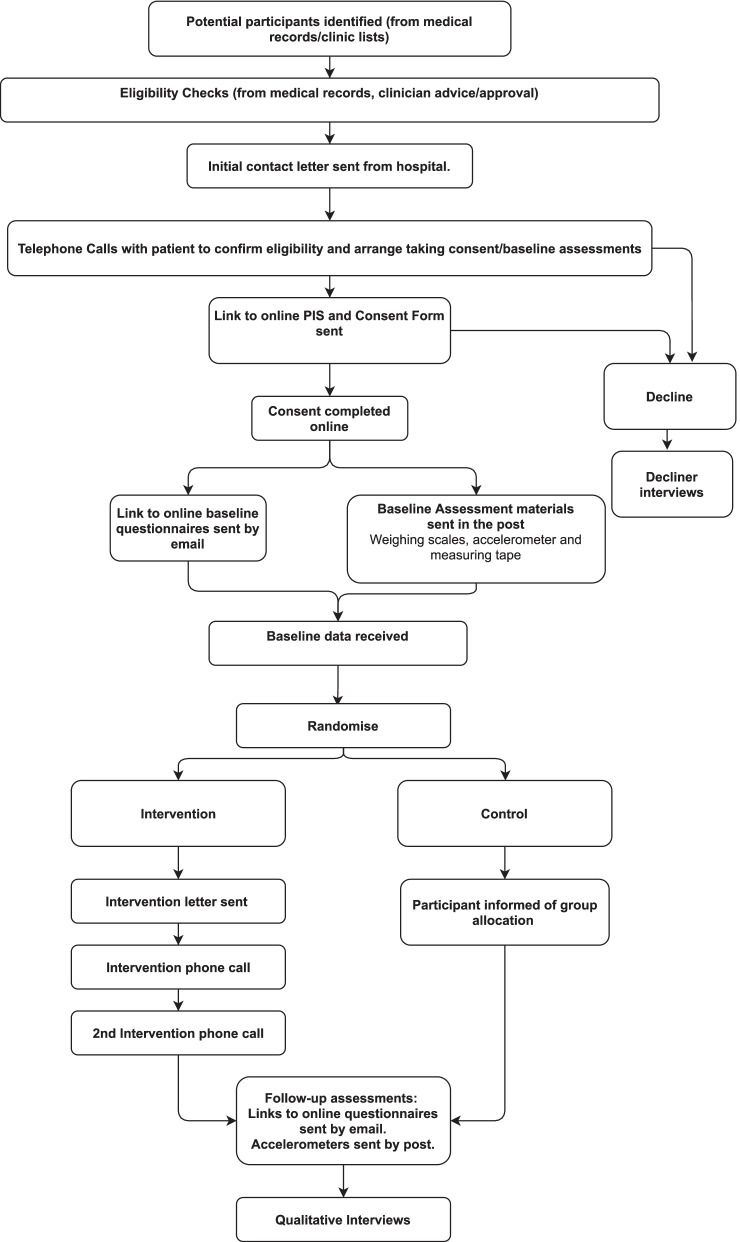
Study flowchart

**Fig. 2:**
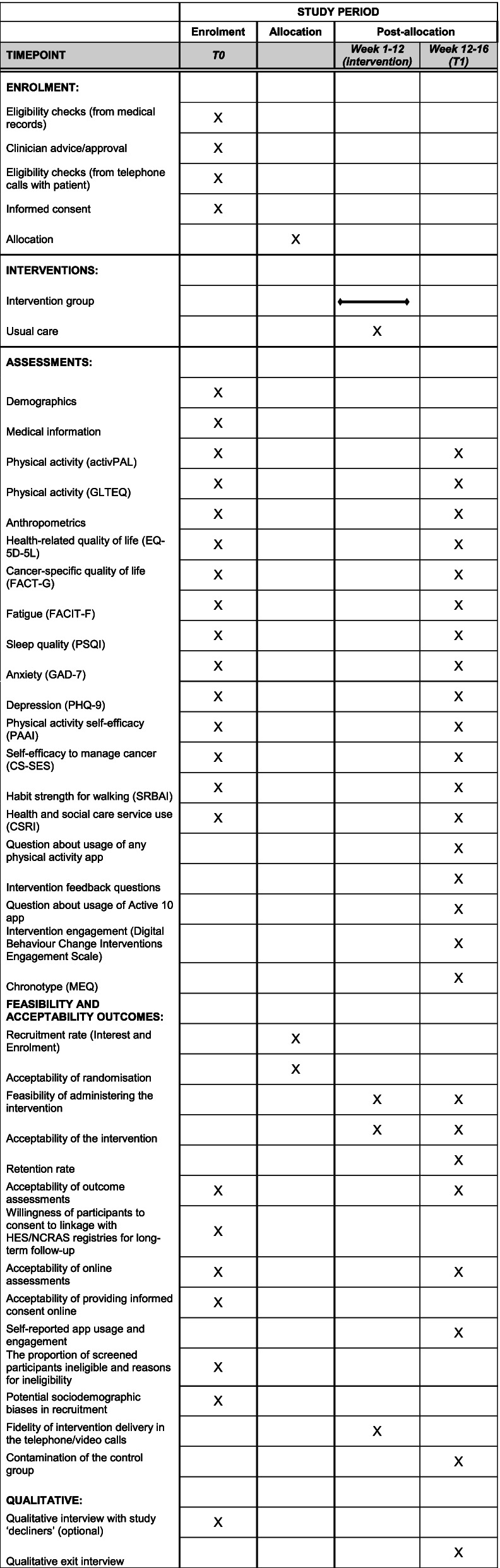
SPIRIT figure—schedule of enrolment, interventions, and assessments

The proposed study is an individually randomised, two-arm pilot RCT comparing an app-based brisk walking intervention delivered alongside standard care, with a control (standard care) arm in people with breast, prostate, or colorectal cancer. The trial has been designed in accordance with the Consolidated Standards of Reporting Trials (CONSORT) statement and its adaptation to pilot trials [31, 32]. See Fig. 1 for a flowchart of the study. The reporting of this protocol follows the Standard Protocol Items: Recommendations for Interventional Trials (SPIRIT) guidelines (see Supplementary Material for a completed SPIRIT checklist) [33] A schedule of enrolment, interventions, and assessments based on the SPIRIT guidelines is shown in Fig. 2.

## Eligibility criteria

Participants will be eligible if they have a confirmed diagnosis of breast, prostate, or colorectal cancer at a single hospital site in South Yorkshire, are aged 16 years or older, own a smartphone that uses Android or iOS (Apple) operating systems, are able to provide informed consent, have access to a computer and an email address, and are willing to complete online questionnaires. This hospital serves an area of high deprivation where cancer incidence, mortality, physical inactivity, and obesity are all high compared to the English average [34, 35]. Participants aged 16 and 17 years of age are not excluded from participation as it has been found that this group are keen to be included in cancer research and have often been overlooked in previous research [36, 37].

Exclusion criteria are as follows: having localised disease and it has been more than 6 months since completion of radical treatment (i.e. surgery to remove cancer, radiotherapy, systemic therapy with curative intent), being unable to understand spoken/written English, having an Eastern Cooperative Oncology Group (ECOG) performance status ≥ 3, a diagnosed cognitive impairment (e.g. dementia), a cognitive and/or physical impairment that prevents participation in brisk walking, a clinician-estimated life expectancy of < 6 months, or are receiving end of life care, due to have surgery to remove cancer in the next 5 months, < 6 weeks after surgery to remove cancer, report already achieving 150 min of at least moderate-intensity physical activity weekly, report previous/current use of the intervention app, or report current or recent (< 6 months) participation in a health behaviour change study.

## Sample size

The target sample size for the pilot RCT is 60, with 30 participants allocated to each group. This is based on the rule of thumb that 30 or more participants are required to estimate a parameter in a feasibility study [38, 39]. A total of 90 participants will be recruited to account for potential loss to follow-up (assuming equal drop-out in both groups).

## Recruitment and setting

Research nurses/members of the research team with contractual arrangements at the hospital site will search lists of current breast, prostate, and colorectal cancer patients and examine medical notes to identify potentially eligible participants. At least one clinician with responsibility for the patient’s care will review the list of potentially eligible participants and confirm whether a patient can be approached about the study.

Potentially eligible participants will be sent a letter informing them about the study. Participants who indicate interest will answer a telephone-based eligibility screening questionnaire. If eligible, participants will be sent an email with a link to the online participant information sheet and consent form (administered via REDCap electronic data capture tools hosted at University College London (UCL)) [40, 41]. The consent form (see Supplementary Material) includes asking for optional additional consent to access Hospital Episode Statistics (HES) and the National Cancer Registration and Analysis Service (NCRAS) registries to understand the willingness to consent to long-term follow-up of medical records. In this pilot, we will not access this data, but we ask participants to consent, as we would in a larger trial, in order to understand willingness to consent to this.

## Randomisation

After completion of baseline assessments, participants will be individually randomised using minimisation with a 1:1 allocation ratio. Randomisation will be undertaken centrally using MinimPy (an open source, minimisation programme for allocation of participants to groups in randomised trials) [42]. Randomisation will be stratified by cancer type (breast, prostate, or colorectal) and disease status (advanced/metastatic disease vs. not). After the first participant has been randomly allocated, each subsequent participant will be allocated to the trial arm with the lowest imbalance score, with the addition of a 20% random element to reduce predictability of outcomes. The imbalance score is calculated based on hypothetical allocation of the next participant to each arm [43].

A member of the research team not involved with recruitment or data collection will use MinimPy to generate the allocation sequence as each person is recruited into the study. Intervention participants will be informed about their allocation arm by letter, with an appointment time for their intervention telephone/video call. Control participants will be informed via telephone or email.

## Feasibility outcomes

The feasibility and acceptability outcomes are described in detail in Table 1 and include the recruitment and retention rates as well as app usage and engagement. These outcomes will be used to assess whether a future definitive trial could continue as per the current protocol, or if revisions are required before moving to the larger trial. The results of a power calculation will be considered alongside the recruitment and retention rates in order to estimate the number of participants that would need to be invited to provide the required sample size, to assess if this is feasible. In addition, if study enrolment is less than 30% or the 3-month retention rate is less than 65% we will consider if the trial procedures need modifying to make them more acceptable. Adaptations will be made to the assessment measures if the results indicate that these were not acceptable to participants or that participants were unable to complete them online. If more than 50% of the intervention participants do not download or use the app, or we are not able to deliver the behaviour change techniques (BCTs) to them as planned then this will trigger discussion about acceptability of the intervention. In lieu of clear published guidelines to base these values on these are pragmatic decisions that will trigger discussion about adaptation of the protocol. As part of the economic evaluation, a value of information analysis will be conducted. Value of information analysis helps ascertain the likely value of obtaining further information and therefore may provide useful information in the context of proceeding to a full RCT. While the listed criteria will be the primary criteria considered we will also examine the results from all data collected and any issue relating to successful trial delivery will inform decisions about progressing to a larger trial.

**Table 1:** Feasibility and acceptability outcomes

Outcome	Measure
Recruitment rate	
Interest	Percentage of those potentially eligible (from medical records and clinician approval) who are interested and willing to answer further eligibility questions.
Enrolment	Percentage of participants who are interested and eligible who are randomised.
Acceptability of randomisation	The percentage of participants who withdraw upon being informed of allocation (within 1 week of randomisation). The percentage of potential participants who state that randomisation is their reason for declining to participate.
Feasibility of administering the intervention	The percentage of the intervention group who: - receive a behavioural support call. - self-report successfully downloading the app.
Acceptability of the intervention	Percentage of participants who report that no aspect of the intervention (leaflet, call, planner, app) was useful. Percentage of withdrawals from intervention group compared to control group. Percentage of reasons for withdrawal relating to the intervention.
Retention rate	The percentage of participants, in each group, who complete any of the T1 follow-up assessment measures.
Acceptability of outcome assessments	The percentage of participants who consent who complete any baseline assessments. Completion rates, in each group, for each of the assessments at: - baseline - follow-up
Willingness of participants to consent to linkage with HES/NCRAS registries for long-term follow-up	The percentage of participants who consent for this aspect of the study.
Acceptability of online assessments	The percentage of participants who require help from a researcher to complete questionnaires. The percentage of potential participants who give this method of data collection as a reason for declining to participate.
Acceptability of providing informed consent online	The percentage of potential participants that state that they are unable/unwilling to provide consent online.
Self-reported app usage and engagement	Percentage of participants in the intervention group who report using the app for less than a month.
The proportion of screened participants ineligible and reasons for ineligibility	The number of participants screened and deemed ineligible for each inclusion/exclusion criteria.
Potential sociodemographic biases in recruitment	Anonymised aggregate socio-demographics (age, gender, ethnicity, Index of Multiple Deprivation, cancer type, cancer stage, time since diagnosis, treatment completed and started) of potentially eligible participants (from medical records and clinician approval) who did not participate in the trial compared with the study sample characteristics.
Fidelity of intervention delivery in the telephone/video calls	Average percentage of required behaviour change techniques covered in 25% of participants’ intervention calls (randomly selected) scored against a checklist.
Contamination of the control group	The percentage of participants in the control group who report: • using Active 10 during the study period. • that a health professional recommended Active 10 to them during the study period.

## Intervention

The intervention is described according to the template for intervention description and replication (TIDiER) checklist which is provided as Supplementary Material [44]. The intervention was designed with input from people affected by breast, prostate, and colorectal cancer throughout, including our background empirical research and several Patient and Public Involvement (PPI) activities [25, 29, 30].

The intervention involves the Active 10 app along with additional behavioural support (outlined in the subsequent sections).

**Fig. 3:**
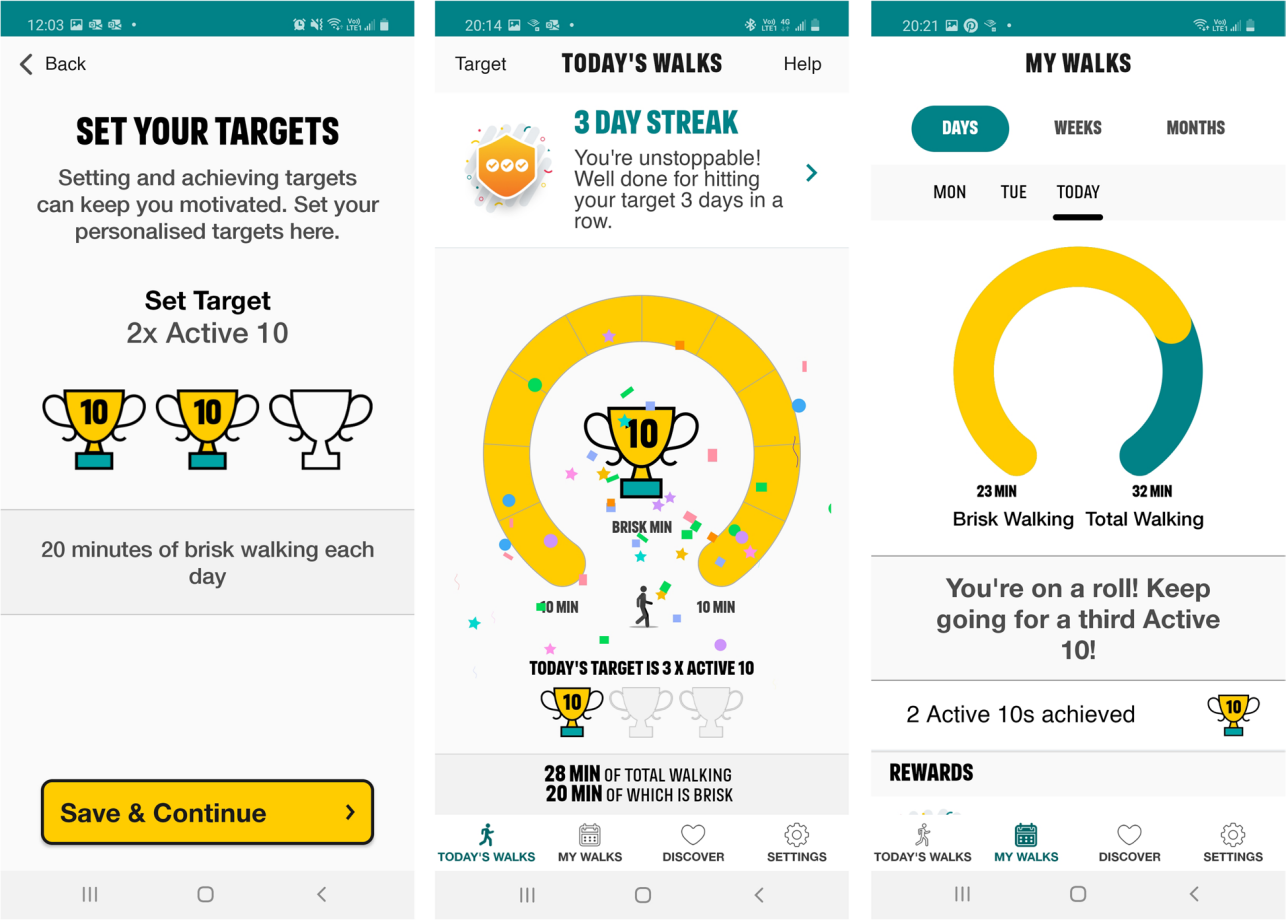
Screenshots of Active 10 app

The Active 10 app was developed by Public Health England and will be maintained by its successor bodies. It was selected for the current study because it contained a number of the features that people LWBC and clinicians highlighted as important and is developed and maintained by a UK health agency, sponsored by the UK Government [29, 30, 45]. Screenshots of the Active 10 app are presented in Fig. 3. The app encourages users to walk briskly for 10 min (known as one ‘Active 10’) and users can set a goal to complete between 1 and 3 Active 10s per day, with the ultimate aim of reaching 30 min of at least moderate-intensity physical activity per day. Each minute of brisk walking counts towards the Active 10 goals, to reflect the recent change in the UK physical activity guidelines removing the guidance that bouts of at least 10 min were required [46]. The app distinguishes between total walking and brisk walking. Brisk walking confers greater health benefit than slower paced walking and is captured by Active 10 when participants walk at a rate of approximately 100 steps per minute or more [47].

Participants will be mailed a pack that contains a leaflet recommending the use of/describing how to download the Active 10 app as well as information about the importance of physical activity after cancer. The pack will also contain walking planners designed to support action planning and self-monitoring of walking plans. These materials will be accompanied by a letter from the participant’s clinical care team endorsing physical activity and using Active 10. Participants will also receive additional behaviour change support from the research team via two telephone/video calls, one shortly after randomisation and a second after 4 weeks. During the initial call researchers will discuss the recommended physical activity guidelines for people LWBC, the associated benefits of meeting these guidelines and of increasing physical activity by any amount; work through the concept of habit formation and using the walking planner; help with setting daily walking goals; help with developing a plan/habit for opening the app; and help with downloading the app for participants who have not already done so. During the second call, researchers will check if participants are using the app and increasing their brisk walking; remind them of their goals; and recap any of the information from the first call. These calls are intended to closely replicate conversations that a health care professional could have with a patient as part of routine care, should this intervention be implemented on a larger scale.

### Theoretical basis of the intervention

Active 10 is a publicly available app and was not developed specifically for this study. Therefore, the app content was independently coded according to the Behaviour Change Technique Taxonomy (BCTTv1) (PL, NM), any discrepancies were discussed before agreement was reached on the techniques used [48]. Table 2 outlines the intervention components across the five elements of the intervention and the relevant coded BCTs [48]. The central feature of the Active 10 app is that it allows participants to monitor their activity. Self-monitoring of physical activity using a variety of technologies has been shown to successfully promote increases in physical activity in the general population and among people LWBC [49, 50]. The intervention content provided in addition to the Active 10 app was informed by habit theory and includes BCTs that have shown efficacy in promoting physical activity in inactive adults, have been associated with improved adherence to physical activity interventions in people LWBC, and that were practical to use in the context of providing brief written materials and behavioural support [28, 51–54].

**Table 2:** Intervention components and the behaviour change techniques

	Intervention component	Behaviour change techniques (BCTTv1)
Active 10 App	Introducing the app into participants’ environment	12.5
	The App is hosted by Public Health England	9.1
	Introduction: “Brisk walking is…” “Every minute counts” “Aim for 10 min or more a day”	4.1
	I’m doing Active 10 because…	N/A
	Set your targets (1, 2, or 3 Active 10s a day)	1.1
	Walking tracker (minutes of walking and minutes of brisk walking)	2.2
	Rewards	10.3, 10.6
	Links to useful websites (e.g. NHS)	N/A
	Link to a discussion group for the app	3.1
	Articles on starting small and building up, physical and mental health benefits, disabilities, how much physical activity to do, and a link to a running app	5.1, 5.6
	Ability to set reminders	7.1
	Tips: social distancing, set a reminder, keep track (use app to see how you’re doing), plan ahead (the day before or in the morning).	N/A
Leaflet and accompanying letter from clinical team	Clinical team recommendation to read and use the information provided and to download Active 10	6.3, 9.1
	Branding: Yorkshire Cancer Research, UCL, University of Leeds, University of Sheffield, Doncaster and Bassetlaw Teaching Hospitals	9.1
	Physical activity improves side effects of cancer treatment, recovery and risk of recurrence, mood and confidence. Physical activity reduces risks of other health problems.	5.1, 5.6
	Quotes from cancer patients: used physical activity to cope with fatigue, chose walking to try to meet guidelines	6.3
	People who have or have had cancer recommended to try to meet same physical activity guidelines as other adults. Brisk walking 2-3 times every day will meet the activity guidelines (150 min), the more the better.	9.1
	Recommends brisk walking. This should make you breathe a bit faster…	4.1
	Recommends start small then build up	N/A
	Information on downloading Active 10	N/A
	Recommends planning	N/A
	Recommends walking at the same time or in the same situation	8.1, 8.3
	Recommends tracking behaviour using the walking planners and Active 10	N/A
	Links to resources about the health benefits of PA	5.1, 5.6
	Links to resources to support walking	6.3
Walking planner	Adding planner to people’s environment	12.5
	Promotes habit formation	8.1, 8.3
	How many Active 10s are you aiming for	1.1
	Plan: when, where, for	1.4, 7.1
	Did you complete plans?	2.3
	Did you meet your target?	2.3
	How did you feel after you walked briskly?	5.4
	Reminder not to worry if miss a day and to adjust goals as required (reduce if finding it hard and increase if meeting goals and feel able).	N/A
Intervention: Phone/video call 1	Introduce self as working with clinical team at the hospital	9.1
	Ask participants how their cancer and treatment has impacted their lifestyle and activity levels	N/A
	Discuss physical and mental health benefits of physical activity	5.1, 5.6
	Discuss motivations to increase activity	9.2
	Discuss concerns about increasing activity	9.2
	Help participant to work out ways to overcome concerns about brisk walking, provide information as appropriate	1.2
	Discuss why recommending brisk walking, including cancer patients have recommended this	6.3
	Describe brisk walking	4.1
	Provide information on government guidelines (150 min MVPA) as well as WHO Every Move Counts.	9.1
	Highlight building up over time	N/A
	Discuss how confident they are and how they can increase their confidence	1.2
	Suggest trying it to see if that increases their confidence	4.4
	If needed tell them that it is possible for them to do this and others have been able to	15.1
	Promote habit formation for initiating a walk	8.1, 8.3
	Make an action plan (when, what, how long)	1.4
	Promote self-reward during and/or after walking	10.7, 10.9
	Promote non-specific self-reward during and/or after walking	10.3, 10.6
	Promote using the app to track activity	2.2
	Promote specific cues	7.1
	Promote reminders	7.1
	Set a target number of Active 10s	1.1
	Promote asking friends to support, by encouraging and helping to remember to walk	3.2, 3.3
	Promote using the walking planner to track behaviour	2.3
	Encourage participants to use information provided to overcome their concerns about exercising	13.2
Intervention: Phone/video call 2	Remind them of their target	1.5
	Ask how they are getting on with their target	1.6
	Ask if they have or, if they want to change their target	1.5
	Ask participants what is preventing them from walking and what would help them to start (if relevant)	1.2
	Repeat any of the points from call 1 as appropriate	As above as relevant

1.1 Goal setting, 1.2 Problem solving, 1.4 Action planning, 1.5 Review behaviour goals, 1.6 Discrepancy between current behaviour and goal, 2.2 Feedback on behaviour, 2.3 Self-monitoring, 3.1 Social support (unspecified), 3.2 Social support (practical), 3.3 Social support (emotional), 4.1 Instruction on how to perform a behaviour, 4.4 Behavioural experiments, 5.1 Information about health consequences, 5.4 Monitoring of emotional consequences. 5.6 Information about emotional consequences, 6.3 Information about others’ approval, 7.1 Prompt/cue, 8.1 Behavioural Practice/Rehearsal, 8.3 Habit formation, 9.1 Credible source, 9.2 Pros and cons, 10.3 Non-specific reward, 10.6 Non-specific incentive, 10.7 Self-incentive, 10.9 Self-reward, 12.5 Adding objects to the environment, 13.2 Framing/Reframing, 15.1 Verbal persuasion about capability

Habit theory posits that habitual behaviours are performed when an impulse to act is automatically triggered in a particular situation by virtue of a mental association having formed between that situation and behaviour through repetition [27]. Habits predict behaviour particularly on days when people’s intentions are lower than usual and therefore support maintenance of behaviour, shielding it from temporary lapses in motivation [55]. In order to form a habit, a behaviour needs to be performed consistently in the same situation (termed context-dependent-repetition) [28]. It is possible to succinctly deliver this advice to participants, and interventions using this technique have shown positive changes in behaviour [56]. Distinction has been made between the habit of initiating a behaviour (i.e. an instigation habit) and that of performing it (i.e. an execution habit), and it is the instigation habit that predicts behaviour maintenance [57–59]. The advice given in the intervention leaflet and phone calls therefore recommend participants focus on forming instigation habits for doing physical activity, while gradually increasing the amount or intensity of activity they do on each occasion.

## Control

Participants randomised to the control group will receive only the study assessments and continue with their standard care. This is so that in the definitive trial the impact of the intervention over and above standard care can be evaluated.

## Measures

Measurement timepoints for the pilot are baseline (T0) and 3 months (T1; operationalised as 12–16 weeks from randomisation). When a participant provides consent they will be sent URL links to online questionnaires via email (with the option to complete these by phone with a researcher if they experience difficulties), and mailed weighing scales (Seca 803 if they weigh less than 150kg and Seca 813 if they weigh over 150kg), a tape measure (Seca 201), and an activPAL4micro accelerometer (PAL Technologies Ltd., Glasgow, UK).

### Sociodemographic & disease characteristics (T0)

Details of each participant’s cancer diagnosis, treatment(s), and other health conditions (including previous cancer diagnoses) will be recorded from their hospital medical notes.

In the online questionnaires, participants will report their age, gender, employment, education, marital status, living arrangements, and ethnicity. Participants will be asked to self-report details of their cancer diagnosis, treatment, and other comorbid health conditions. This is to capture any further health information that is not included in the hospital medical records (e.g. information that would otherwise be included in GP records, or records from other hospitals). Participants’ postcodes will be used to determine socioeconomic position (Index of Multiple Deprivation) [60].

### Physical activity (T0 and T1)

Physical activity will be measured using thigh-worn activPAL4micro accelerometers that participants will be asked to wear continuously for seven days (PAL Technologies Ltd., Glasgow, UK). The activPAL protocol, which follows published recommendations for using the device and expert advice from the Trial Steering Committee, includes waterproofing the device using specially designed nitrile sleeves and waterproof dressings, asking participants to wear it continuously, and including the data for analysis if 3 days of data are available [61]. Participants will be mailed instructions on how to wear the device, a log-sheet to record when the device was worn and bedtimes and waketimes, and a freepost envelope to return it. The primary outcome of the future full RCT will be activPAL-assessed average daily brisk walking (> 100 steps/min). The activPAL has shown excellent reliability and validity in measuring step number and cadence (steps/minute) and has been used in other studies with people LWBC and clinical populations [62, 63]. Other physical activity outcomes that will be explored are total daily steps, minutes of light physical activity, standing time and sitting time.

Participants will also complete the Godin Leisure-Time Exercise Questionnaire (GLTEQ) [64], which has demonstrated favourable validity and reliability against objective measures of physical activity and is widely used in oncology research [64, 65]. The questionnaire will be adapted to add a question about duration of activities to allow calculation of minutes of MVPA, in addition to the leisure score index, a practice that is common in oncology research [65].

### Anthropometric outcomes (T0 and T1)

Participants’ height, weight (without outer clothing/shoes on) and waist circumference (at umbilicus) will be measured by participants in their own homes using the study weighing scales and measuring tapes provided. Written instructions will be included to help participants complete these measurements accurately. Studies suggest that self-reported weight is sufficiently reliable and accurate where objective measurement is not feasible [66, 67]. BMI will be calculated using the standard formula of weight (kg)/height (m)^2^. Self-measured waist circumference is also appropriate for large-scale studies where objective measurement is not feasible [68].

### Well-being (T0 and T1)

Health status will be measured using the five-level EuroQol-5D questionnaire (EQ-5D-5L), which has established reliability and validity from a review of 12 studies of cancer patients [69]. This will be used to generate quality-adjusted life years (QALYs) to facilitate the cost-effectiveness analysis.

Cancer-specific quality of life will be measured using the Functional Assessment of Cancer Therapy-General (FACT-G) scale [70]. The FACT-G is a 28-item questionnaire that has excellent test-retest reliability (*r* = .92) and has been validated against the Functional Living Index-Cancer (FLIC) (*r* = .79) [70, 71].

Fatigue will be measured using the 13-item fatigue subscale of the Functional Assessment of Chronic Illness Therapy-Fatigue (FACIT-F) (previously Functional Assessment of Cancer Therapy-Fatigue (FACT-F)) questionnaire [72]. The 13-item fatigue subscale of the FACIT-F has excellent test-retest reliability (*r* = .90), internal consistency (*α* = .93–.95), and has been validated against the Profile of Mood States (POMS) fatigue (*r* = − .74), POMS vigour (*r* = .66) and Piper fatigue (*r* = − .75) [72–74].

Sleep quality will be assessed using the Pittsburgh Sleep Quality Index (PSQI), an 18-item questionnaire that assesses sleep quality and disturbances over a 1-month time interval [75]. The PSQI has been used extensively and has good psychometric properties in both clinical (including cancer) and non-clinical samples [76]. In women with breast cancer, the PSQI has been demonstrated to have high internal consistency (*α* = .80) and good validity when compared against related constructs such as sleep problems (in the Symptom Experience Report; *r* = .65) and sleep restlessness (in the Center for Epidemiological Studies Depression Scale; *r* = .69) [77].

Anxiety will be measured with the Generalised Anxiety Disorder Assessment (GAD-7), which has good test-retest reliability (*r* = .83), excellent internal consistency (*α* = .92), and has been validated against the Beck Anxiety Inventory (*r* = .72) [78]. Depression will be measured with the Patient Health Questionnaire (PHQ-9), which shows adequate diagnostic accuracy in cancer patients [79–81].

Physical activity self-efficacy will be measured using the Physical Activity Appraisal Inventory (PAAI), which has demonstrated excellent reliability in women with breast cancer (*α* = .96) [82]. The PAAI also has established validity [82]. Self-efficacy to self-manage cancer will be measured using the Cancer Survivors Self-Efficacy Scale (CS-SES), an 11-item questionnaire with excellent reliability (*α* = .92) [83].

Habit strength for walking (‘going for a walk’ and ‘walking briskly’) will be measured with the Self-Report Behavioural Automaticity Index (SRBAI), which has established reliability and has shown to be sensitive to hypothesised effects of habit on behaviour [84].

Health and social care service use will be measured using the Client Service Receipt Inventory (CSRI), which has been validated against objective primary care records and is also recommended for usage of hospital and other community health services [85].

### App use (T1)

Participants in both groups will also be asked to report their usage of any physical activity app, or any other attempts to change their physical activity, during the study period, and asked what prompted them to do this. Intervention group participants will be asked to complete brief intervention feedback questions (including a self-report question of whether they ever downloaded Active 10) and will be asked to self-report their usage of Active 10 throughout the study period (never, once, less than monthly, monthly, fortnightly (every 2 weeks), weekly, 3–4 times per week, almost every day or every day). Intervention participants will also be asked to complete questions from the Digital Behaviour Change Interventions Engagement Scale [86].

### Timing of physical activity (T1)

In a definitive trial, we would conduct an exploratory analysis using the activPAL data alongside habit strength to investigate whether those who walk in the morning have higher habit strength than those who walk in the evening. Previous research suggests that habits form quicker in the morning than the evening [87]. Chronotype (individual differences in sleep timing and in preferences for a given time of day) will be considered a covariate in this analysis and assessed using a sub-scale of the Morningness-Eveningness questionnaire (MEQ) [88, 89]. This sub-scale has been found to be reliable and to correlate well with the full scale [90].

### Qualitative interviews (decliners at T0 and participants at T1)

To further understand how a future intervention could be designed to be as inclusive as possible, individuals who decline to participate will be asked to briefly provide reasons if they are willing. They will also have the option to consent to participate in an interview to further explore reasons, and up to 30 interviews will be conducted. This method has been utilised succesfully in an ongoing exercise trial with myeloma patients [63].

Interviews will also be carried out at the end of the trial with any participants who agree to be interviewed. The interviews will take place after a participant has completed all other data collection at T1 and will be conducted to explore experiences of participation, being randomised, and views on providing permission to access NCRAS/HES data. Intervention arm participants will also be interviewed about their experiences of using the app, the intervention materials, and their perceptions of app usage and engagement. Participants will be invited to submit photographs of the places that they walk. This is optional, but if participants consent, they will be able to upload pictures to a secure website along with brief details of where the photograph was taken, why they were walking there, how long they walked and any additional details they wish to share. Photographs will be used to prompt discussion about the environments chosen for brisk walking. This method of photo-elicitation has been used to understand walking environments in previous work and led to a more in-depth understanding of barriers and facilitators to walking [91]. All interviews will be semi-structured and based on a topic guide, take place via telephone, and will be audio-recorded and transcribed verbatim.

## Statistical analysis

Baseline comparability of the randomised groups will be assessed using descriptive statistics (e.g. age (continuous), gender (categorical), key outcome measures (e.g. physical activity participation)). The outcomes outlined in Table 1 will be reported descriptively. Any reasons provided for declining not covered in the specific outcomes will also be reported, as well as details of answers provided to the feedback questionnaire, within the intervention group. Mean and standard deviation estimates for the intended primary outcome measure at 3 months (activPAL measured average daily minutes of brisk walking (> 100 steps/min)) will be calculated and reported descriptively. These results will be used to inform a sample size calculation for the future definitive trial.

An initial economic evaluation will be conducted to provide an estimate of the potential cost-effectiveness of the intervention. QALYs will be estimated based upon the EQ-5D-5L combined with standard valuation sources [92, 93]. Costs will include the costs associated with the intervention (promotional materials, time spent delivering the intervention recommendation) and other NHS resource use measured using the CSRI. Costs and QALYs will be combined in an analysis to estimate the incremental cost-effectiveness ratio (ICER) associated with the intervention compared to the control group. Uncertainty in the results will be characterised using cost-effectiveness planes and cost-effectiveness acceptability curves [94]. A value of information analysis will also be conducted at this stage. The expected value of perfect information (EVPI) and expected value of perfect partial information (EVPPI) will be estimated.

## Qualitative analysis

Qualitative interviews will be analysed thematically using an approach that is both deductive and inductive, to ensure that the full range of participants' responses are represented, following the steps outlined by Braun and Clarke [95]. These findings will add more in-depth understanding to the feasibility and acceptability analysis described above.

## Ethical considerations

The trial protocol has been approved by the Yorkshire & The Humber - South Yorkshire Research Ethics Committee (21/YH/0029) and by the Health Research Authority. The Research and Development Department at the study site has also authorised the study to go ahead following a capacity and capability review. Potential amendments to the protocol will only be implemented if ethical and regulatory approval, including NHS permission where required, is obtained.

### Consent

Consent from each participant prior to participation in the trial will be collected online through REDCap hosted within UCL’s Data Safe Haven (details below) [96]. All participants will be informed that they are under no obligation to enter the trial. All participants will also be informed that they can withdraw at any time during the trial without having to give a reason, and that withdrawal will not affect the medical care they receive.

### Confidentiality

This study has been registered for Data Protection at UCL Records Office (Reference: Z6364106/2020/10/29). All data will be handled in accordance with the General Data Protection Regulation (2018) and the UK Data Protection Act (2018). Personal data will only be collected if it is deemed essential for the study. Wherever possible, personal data that has been collected will be pseudoanonymised. No identifiable personal data will be used in dissemination of the research.

### Adverse event reporting

All adverse events (AEs) and serious AEs (SAEs) that the research team become aware of will be assessed for severity, causality, and seriousness. All AEs and SAEs will be recorded. All SAEs will be reported to the sponsor. Events that are unexpected and thought to be related to the intervention will be reported to the Health Research Authority. In this population, a range of SAEs would be expected that relate to their cancer diagnosis and treatment, including episodes of acute illness, infection, new medical problems, and deterioration of existing medical problems. These could result in hospitalisation, permanent disability or incapacity, or death. These would not however be related to the intervention. There is one potential expected AE related to participation in the study; participants might experience mild skin irritation from the adhesive dressing when wearing the activPAL device as this has been observed previously in older adults [97].

### Data monitoring

The sponsor of this trial is UCL. A Trial Management Group (TMG) consisting of the Chief Investigators (AF and PL) and research staff employed on the grant (FK and CM) will be responsible for overseeing the trial. An external Trial Steering Committee, including two independent members, the trial co-investigators, and a lay representative will meet once-twice per year (alongside the TMG) and will provide overall supervision of the trial. There will be no formal data monitoring committee and no criteria have been set for stopping the study early as this is a pilot study and walking is a very low-risk intervention.

### Data management

All study data will be stored securely within the UCL Data Safe Haven encrypted platform [91]. The Data Safe Haven is built using a walled garden approach where the data is stored, processed, and managed within a secure environment [91]. Only members of the research team will be able to access the dataset.

### Data archiving

All electronic research data will be stored securely within the UCL Data Safe Haven for 12 years after the trial end date, after which point the data will be completely anonymised (by removing the study pseudonym and deleting all contact details including proof of consent) [91]. The anonymised dataset will be entered into the UCL Data Repository and available here for at least 20 years from the trial end date. Other study-related documents will be archived at UCL and each participating site for 20 years from the trial end date and in line with all relevant legal and statutory requirements.

### Dissemination

The results of this study will be disseminated through peer-reviewed publications and conference presentations. The results will also be disseminated through social media outlets such as Twitter, and via our PPI representatives.

## Discussion

Conducting a pilot and assessing the feasibility of study procedures is an essential part of developing and evaluating complex interventions [98]. This pilot study follows recommendations to use both quantitative and qualitative measures to assess the feasibility and acceptability of the study design in as much detail as possible [98]. The definitive trial that will be run following revision to the current methodology, as required, will address the limited evidence base for theory-driven smartphone app-based interventions designed to promote physical activity in people LWBC. Yorkshire Cancer Research have awarded our group a grant to complete this pilot study and a larger trial, meaning that the definitive trial will be able to proceed quickly once the results of this pilot have been analysed.

The strengths of the intervention include its tailored and theoretically-informed approach to promote brisk walking, an activity that is perceived as safe, achievable, enjoyable, and sustainable by people LWBC [29]. The design of the intervention has been informed based on input from people LWBC. This intervention is accessible, low cost, and scalable, due to the use of an app maintained by Public Health England (or its successor bodies). It is hoped that the intervention could be replicated by a HCP as part of routine care for people LWBC, should it be implemented on a larger scale. A strength of the trial design is the use of objective physical activity measurement.

A limitation of the intervention is that it is only accessible to participants who own a smartphone. However, this intervention would be most appropriate for those who already have a smartphone and as these rates are increasing, this is becoming the majority of the population [23, 24]. We will collect data on how many participants are excluded because they do not own a smartphone so that this can be addressed in a future RCT if required. Another limitation is that the study is taking place in one hospital site in South Yorkshire, and thus it may be that the feasibility results do not generalise to all hospital sites. However, as this hospital is in a deprived area recruitment rates would likely be similar or better in other areas. Also, as the recruitment approach does not involve face-to-face contact with participants it is likely transferable across sites. It is possible that the intervention may be more or less acceptable in this area than others; however, if it is acceptable here, this would imply that it is acceptable to those most in need.

As the Active 10 app is publicly available it is possible that those in the control group could use it during the study. However, the intervention is a package that includes a recommendation to use this app alongside behavioural support to do so. Therefore, even if a control group participant does start using the app this is not equivalent to being in the intervention group. Patients have reported that they are keen for health professionals to recommend an app to them [29] therefore we think it unlikely that many of the control group will seek out the app on their own. We will assess this as a feasibility outcome.

To conclude, this pilot RCT will provide information regarding the feasibility and acceptability of testing this intervention to inform a future definitive RCT. The trial will also provide estimates of the parameters of the intended primary outcome measure for a sample size calculation for a future trial. Should the intervention be feasible, with or without adaptations, a future definitive RCT will aim to investigate the clinical and cost-effectiveness of the intervention among people affected by cancer.

## Supplementary Information

**Additional file 1.**

**Additional file 2.**

## Data Availability

No data is reported in this protocol. The full intervention materials are not supplied with the protocol paper since recruitment for the pilot had not started at the time of paper submission and we do not want our materials to be available in the public domain until the end of the trial.

## Abbreviations

AE: Adverse event
BCT: Behaviour change technique
BMI: Body mass index
CONSORT: Consolidated Standards of Reporting Trials
CSRI: Client Service Receipt Inventory
CS-SES: Cancer Survivors Self-Efficacy Scale
ECOG: Eastern Cooperative Oncology Group
EQ-5D-5L: Five-level EuroQol-5D
EVPI: Expected value of perfect information
EVPPI: Expected value of perfect partial information
FACIT-F: Functional Assessment of Chronic Illness Therapy-Fatigue
FACT-F: Functional Assessment of Cancer Therapy-Fatigue
FACT-G: Functional Assessment of Cancer Therapy-General
FLIC: Functional Living Index-Cancer
GAD-7: Generalised Anxiety Disorder Assessment
GLTEQ: Godin Leisure-Time Exercise Questionnaire
HCP: Healthcare professional
HES: Hospital Episode Statistics
ICER: Incremental cost-effectiveness ratio
LWBC: Living with and beyond cancer
MVPA: Moderate-to-vigorous physical activity
NCRAS: National Cancer Registration and Analysis Service
PAAI: Physical Activity Appraisal Inventory
PHQ-9: Patient Health Questionnaire-9
POMS: Profile of Mood States
PPI: Patient and Public Involvement
PSQI: Pittsburgh Sleep Quality Index
QALY: Quality-adjusted life year
RCT: Randomised controlled trial
SAE: Serious adverse event
SPIRIT: Standard Protocol Items: Recommendations for Interventional Trials
SRBAI: Self-Report Behavioural Automaticity Index
TIDieR: Template for intervention description and replication
TMG: Trial Management Group
UCL: University College London
