# Supplementary material for: An app with brief behavioural support to promote physical activity after a cancer diagnosis (APPROACH): study protocol for a pilot randomised controlled trial
Source: Pilot Feasibility Stud. 2022 Mar 29;8:74. doi: 10.1186/s40814-022-01028-w (PMC8961486; doi:10.1186/s40814-022-01028-w)
Supplement: Supplementary file 1 — Additional file 1. [file 40814_2022_1028_MOESM1_ESM.docx]

**Supplementary Material 1**

**TIDiER checklist**

| **Name** | APPROACH: app-based intervention with brief behavioural support to promote physical activity in people affected by cancer |
| --- | --- |
| **Why** | Physical activity has been shown to improve multiple physical and psychosocial outcomes after a cancer diagnosis. Thus, people living with and beyond cancer (LWBC) are recommended by the World Cancer Research Fund to engage in ≥150 minutes of at least moderate intensity physical activity per week. However, many people LWBC do not meet these physical activity guidelines. Many people LWBC report receiving little information about physical activity from healthcare professionals, including oncologists. Healthcare professionals report many barriers to giving physical activity advice, including lack of knowledge of guidelines, not feeling like the right person to give advice, and lack of time and resources [41-44]. Thus, physical activity interventions are needed that could be rolled out to many people LWBC and that could be delivered by healthcare professionals with low cost and ease. Smartphone apps provide a promising platform for scalable behaviour change interventions. However, there is limited research exploring the potential of smartphone apps in physical activity promotion after cancer. Qualitative interview studies with people LWBC have shown that they feel favourably towards apps that promote walking and feel that it is important that such apps are endorsed by cancer clinical nurse specialists. Interviews with cancer clinical nurse specialists have shown that they also feel positive about the role of walking-based apps for people LWBC.  Therefore, the proposed study aims to (1) investigate the feasibility and acceptability of the procedures and design of an app-based intervention with brief behavioural support to promote physical activity in people affected by cancer and (2) provide estimates of the parameters for the intended primary outcome measure (activPAL measured brisk walking (>100 steps/minute)) for the sample size calculation for the future definitive RCT. The study will be a pilot RCT and will be conducted in South Yorkshire in the UK. |
| **What (materials)** | The intervention group will be recommended to use Active 10, an app developed by Public Health England that will be maintained by its successor bodies. The Active 10 app enables the setting of goals (e.g. 1 to 3 Active 10s per day), provides feedback on the number of minutes walked briskly per day, gives information about the health and emotional consequences of physical activity, enables the setting of reminders to walk, and provides non-specific rewards if walking targets have been met.  Participants in the intervention group will also be given a leaflet containing information about physical activity and cancer and walking planners to support active planning and monitoring of their walking plans. Participants will receive additional behaviour change support from the research team via two telephone/video calls, one occuring at baseline and one at four weeks. The content of these materials are based on Habit Theory and include behaviour change techniques which have shown efficacy in promoting physical activity in inactive adults. These calls are intended to closely replicate conversations that a healthcare professional (e.g. Clinical Nurse Specialist) could have with a patient as part of routine care, should this intervention be implemented on a larger scale. |
| **What (procedure)** | Upon randomisation to the intervention group, intervention participants will be posted a letter of endorsement of physical activity and using the app from their clinical team, and the intervention materials (leaflet and walking planners), with an appointment time for a researcher to call and deliver the intervention telephone/video call. The materials will encourage participants to download the Active 10 app in advance of the call with the researcher.  During the first telephone/video call, the researchers will discuss the recommended physical activity guidelines for people LWBC; the associated benefits of meeting these guidelines and of increasing physical activity by any amount; work through the walking planner; help with setting daily walking goals; help with developing a plan/habit for opening the app; and help with downloading the app for participants who have not already done so. After the call, participants will be free to use the Active 10 app, leaflet and walking planners at their own discretion. Participants will receive a second call at 4 weeks to check how they are getting on; to remind them of their goals; and to re-cap any of the information from the first call. |
| **Who provides** | The study researchers, who have extensive research experience in working with people LWBC, will provide participants with behavioural change support in the telephone/video calls. |
| **How** | Upon randomisation to the intervention group, intervention participants will be posted a letter of endorsement of physical activity and using the Active 10 app from their clinical team, and the intervention materials, consisting of the leaflet and walking planners. Participants will receive a one-to-one telephone/video call at baseline and at four weeks for additional behaviour change support. Participants will access the Active 10 app via their smartphone. |
| **Where** | The intervention will be delivered remotely and participants will be able to access all the intervention elements at a location of their choice. |
| **When and how much** | Frequency of using the Active 10 app, walking planners and leaflet will be at the discretion of the participant. Participants will receive a telephone/video call at baseline and 4 weeks. |
| **Tailoring** | All participants will receive the same intervention materials: Active 10 app, leaflet and walking planner. However, participants can choose their own Active 10 goals, which will be set in the baseline telephone/video call. These goals can be adapted at any time and will be discussed during the follow-up telephone/video call if participants are not meeting their goals and want to decrease them, or if they are meeting or exceeding their goals and want to increase them. During the telephone calls, and independently, participants will have the opportunity to make their own plans for when and how they will add brisk walking to their days. |
| **How well** | Fidelity of intervention delivery in the telephone/video calls will be assessed by scoring the recordings of the intervention calls against a checklist of content, including the behaviour change techniques included in the script, to assess how many of these techniques were covered. This could inform adaptation of the script ahead of a larger trial, as required. |

**SPIRIT 2013 Checklist**

| Section/item | Item No | Description | Addressed |
| --- | --- | --- | --- |
| **Administrative information** | | |  |
| Title | 1 | Descriptive title identifying the study design, population, interventions, and, if applicable, trial acronym | ✓ |
| Trial registration | 2a | Trial identifier and registry name. If not yet registered, name of intended registry | ✓ |
|  | 2b | All items from the World Health Organization Trial Registration Data Set | ✓ |
| Protocol version | 3 | Date and version identifier | N/A |
| Funding | 4 | Sources and types of financial, material, and other support | ✓ |
| Roles and responsibilities | 5a | Names, affiliations, and roles of protocol contributors | ✓ |
|  | 5b | Name and contact information for the trial sponsor | ✓ |
|  | 5c | Role of study sponsor and funders, if any, in study design; collection, management, analysis, and interpretation of data; writing of the report; and the decision to submit the report for publication, including whether they will have ultimate authority over any of these activities | ✓ |
|  | 5d | Composition, roles, and responsibilities of the coordinating centre, steering committee, endpoint adjudication committee, data management team, and other individuals or groups overseeing the trial, if applicable (see Item 21a for data monitoring committee) | ✓ |
| Introduction |  |  |  |
| Background and rationale | 6a | Description of research question and justification for undertaking the trial, including summary of relevant studies (published and unpublished) examining benefits and harms for each intervention | ✓ |
|  | 6b | Explanation for choice of comparators | ✓ |
| Objectives | 7 | Specific objectives or hypotheses | ✓ |
| Trial design | 8 | Description of trial design including type of trial (eg, parallel group, crossover, factorial, single group), allocation ratio, and framework (eg, superiority, equivalence, noninferiority, exploratory) | ✓ |
| Methods: Participants, interventions, and outcomes | | |  |
| Study setting | 9 | Description of study settings (eg, community clinic, academic hospital) and list of countries where data will be collected. Reference to where list of study sites can be obtained | ✓ |
| Eligibility criteria | 10 | Inclusion and exclusion criteria for participants. If applicable, eligibility criteria for study centres and individuals who will perform the interventions (eg, surgeons, psychotherapists) | ✓ |
| Interventions | 11a | Interventions for each group with sufficient detail to allow replication, including how and when they will be administered | ✓ |
|  | 11b | Criteria for discontinuing or modifying allocated interventions for a given trial participant (eg, drug dose change in response to harms, participant request, or improving/worsening disease) | N/A |
|  | 11c | Strategies to improve adherence to intervention protocols, and any procedures for monitoring adherence (eg, drug tablet return, laboratory tests) | N/A |
|  | 11d | Relevant concomitant care and interventions that are permitted or prohibited during the trial | N/A |
| Outcomes | 12 | Primary, secondary, and other outcomes, including the specific measurement variable (eg, systolic blood pressure), analysis metric (eg, change from baseline, final value, time to event), method of aggregation (eg, median, proportion), and time point for each outcome. Explanation of the clinical relevance of chosen efficacy and harm outcomes is strongly recommended | ✓ |
| Participant timeline | 13 | Time schedule of enrolment, interventions (including any run-ins and washouts), assessments, and visits for participants. A schematic diagram is highly recommended (see Figure) | ✓ |
| Sample size | 14 | Estimated number of participants needed to achieve study objectives and how it was determined, including clinical and statistical assumptions supporting any sample size calculations | ✓ |
| Recruitment | 15 | Strategies for achieving adequate participant enrolment to reach target sample size | ✓ |
| **Methods: Assignment of interventions (for controlled trials)** | | |  |
| Allocation: |  |  |  |
| Sequence generation | 16a | Method of generating the allocation sequence (eg, computer-generated random numbers), and list of any factors for stratification. To reduce predictability of a random sequence, details of any planned restriction (eg, blocking) should be provided in a separate document that is unavailable to those who enrol participants or assign interventions | ✓ |
| Allocation concealment mechanism | 16b | Mechanism of implementing the allocation sequence (eg, central telephone; sequentially numbered, opaque, sealed envelopes), describing any steps to conceal the sequence until interventions are assigned | N/A |
| Implementation | 16c | Who will generate the allocation sequence, who will enrol participants, and who will assign participants to interventions | ✓ |
| Blinding (masking) | 17a | Who will be blinded after assignment to interventions (eg, trial participants, care providers, outcome assessors, data analysts), and how | ✓ |
|  | 17b | If blinded, circumstances under which unblinding is permissible, and procedure for revealing a participant’s allocated intervention during the trial | N/A |
| **Methods: Data collection, management, and analysis** | | |  |
| Data collection methods | 18a | Plans for assessment and collection of outcome, baseline, and other trial data, including any related processes to promote data quality (eg, duplicate measurements, training of assessors) and a description of study instruments (eg, questionnaires, laboratory tests) along with their reliability and validity, if known. Reference to where data collection forms can be found, if not in the protocol | ✓ |
|  | 18b | Plans to promote participant retention and complete follow-up, including list of any outcome data to be collected for participants who discontinue or deviate from intervention protocols | ✓ |
| Data management | 19 | Plans for data entry, coding, security, and storage, including any related processes to promote data quality (eg, double data entry; range checks for data values). Reference to where details of data management procedures can be found, if not in the protocol | ✓ |
| Statistical methods | 20a | Statistical methods for analysing primary and secondary outcomes. Reference to where other details of the statistical analysis plan can be found, if not in the protocol | ✓ |
|  | 20b | Methods for any additional analyses (eg, subgroup and adjusted analyses) | N/A |
|  | 20c | Definition of analysis population relating to protocol non-adherence (eg, as randomised analysis), and any statistical methods to handle missing data (eg, multiple imputation) | N/A |
| **Methods: Monitoring** | | |  |
| Data monitoring | 21a | Composition of data monitoring committee (DMC); summary of its role and reporting structure; statement of whether it is independent from the sponsor and competing interests; and reference to where further details about its charter can be found, if not in the protocol. Alternatively, an explanation of why a DMC is not needed | ✓ |
|  | 21b | Description of any interim analyses and stopping guidelines, including who will have access to these interim results and make the final decision to terminate the trial | N/A |
| Harms | 22 | Plans for collecting, assessing, reporting, and managing solicited and spontaneously reported adverse events and other unintended effects of trial interventions or trial conduct | ✓ |
| Auditing | 23 | Frequency and procedures for auditing trial conduct, if any, and whether the process will be independent from investigators and the sponsor | N/A |
| Ethics and dissemination | | |  |
| Research ethics approval | 24 | Plans for seeking research ethics committee/institutional review board (REC/IRB) approval | ✓ |
| Protocol amendments | 25 | Plans for communicating important protocol modifications (eg, changes to eligibility criteria, outcomes, analyses) to relevant parties (eg, investigators, REC/IRBs, trial participants, trial registries, journals, regulators) | ✓ |
| Consent or assent | 26a | Who will obtain informed consent or assent from potential trial participants or authorised surrogates, and how (see Item 32) | ✓ |
|  | 26b | Additional consent provisions for collection and use of participant data and biological specimens in ancillary studies, if applicable | ✓ |
| Confidentiality | 27 | How personal information about potential and enrolled participants will be collected, shared, and maintained in order to protect confidentiality before, during, and after the trial | ✓ |
| Declaration of interests | 28 | Financial and other competing interests for principal investigators for the overall trial and each study site | N/A |
| Access to data | 29 | Statement of who will have access to the final trial dataset, and disclosure of contractual agreements that limit such access for investigators | ✓ |
| Ancillary and post-trial care | 30 | Provisions, if any, for ancillary and post-trial care, and for compensation to those who suffer harm from trial participation | N/A |
| Dissemination policy | 31a | Plans for investigators and sponsor to communicate trial results to participants, healthcare professionals, the public, and other relevant groups (eg, via publication, reporting in results databases, or other data sharing arrangements), including any publication restrictions | ✓ |
|  | 31b | Authorship eligibility guidelines and any intended use of professional writers | N/A |
|  | 31c | Plans, if any, for granting public access to the full protocol, participant-level dataset, and statistical code | ✓ |
| Appendices |  |  |  |
| Informed consent materials | 32 | Model consent form and other related documentation given to participants and authorised surrogates | ✓ |
| Biological specimens | 33 | Plans for collection, laboratory evaluation, and storage of biological specimens for genetic or molecular analysis in the current trial and for future use in ancillary studies, if applicable | N/A |
