# Supplementary material for: An app with brief behavioural support to promote physical activity after a cancer diagnosis (APPROACH): study protocol for a pilot randomised controlled trial
Source: Pilot Feasibility Stud. 2022 Mar 29;8:74. doi: 10.1186/s40814-022-01028-w (PMC8961486; doi:10.1186/s40814-022-01028-w)
Supplement: Supplementary file 2 — Additional file 2. [file 40814_2022_1028_MOESM2_ESM.docx]

**ONLINE CONSENT FORM**

***Study title*: APPROACH – an app for health & wellbeing after cancer**

**Would you like to:**

1. **Participate in the APPROACH Study**
2. **Decline to participate in the APPROACH study but would be happy to be interviewed about your reasons for this choice**

**NEXT**

***If chose 1 branching sends to page 3***

***If chose 2 branching sends to page 2***

| **CONSENT FORM for Decliners Interview**  ***Study title*: APPROACH – an app for health & wellbeing after cancer**  **We would like to gather information from people who have been approached to take part in this study, but choose not to participate, to explore their reasons for this choice. If you do not consent to take part in the study but are willing to share your reasons with the study team during a short telephone interview (the interview will be recorded and then transcribed by a specialist external transcription company (*company name*)), please enter your details below. Please note that this it is completely up to you if you would like to do this or not.** |
| --- |

Name

Date

Signature

Telephone number

Email address

Home address

On the next page you will be shown a PDF of all the information you have read here and your responses. You will be able to download this for your records if you wish. If you would also like us to email you a copy of this PDF please click here. ***Radio button***

**NEXT**

*Page break*

*PDF of the information sheet and consent responses will be shown on the screen and participants can download or print this.*

*Participants then confirm they are happy with their responses and submit this form.*

**CONSENT FORM**

***Study title*: APPROACH – an app for health & wellbeing after cancer**

If you would like to take part in the APPROACH study please confirm your agreement with the statments below. For statements where there is the option to respond yes or no, this aspect of the study is optional and you will still be able to participate if you select no. You need to agree to all the other statements to be able to participate.

| 1. I confirm that I have read and understand the information sheet, V1 01/03/2021 for the above study and have had the opportunity to ask questions. | ***Radio button*** |
| --- | --- |
| 1. I understand that my participation in this trial is voluntary and that I am free to withdraw at any time, without giving any reason, and without my medical care or legal rights being affected. | ***Radio button*** |
| 1. I understand that relevant sections of my hospital medical notes may be looked at by individuals from the relevant NHS trusts, and data collected during the study may be looked at by members of the APPROACH research team. Personally identifiable information will only be accessed by individuals from UCL, University of Leeds, or Doncaster and Bassetlaw Teaching Hospitals NHS Foundation Trust and regulatory authorities who need access to this information to conduct the study. | ***Radio button*** |
| 1. I understand that information collected about me may be used to support other research in the future, and may be shared anonymously with other researchers. I will not be identified. | ***Radio button*** |
| 1. I agree to measuring my height, weight and waist circumference and entering this information into an online questionnaire, to completing online questionnaires, and to wearing an activPAL for 7 days on 2 occasions. | ***Radio button*** |
| 1. I agree to being invited to participate in a semi-structured telephone interview at the end of the study and that if I choose to do this, that the interview will be recorded and then transcribed by a specialist external transcription company (*company name*). This is optional and I do not have to agree to be interviewed. | ***Radio button*** |
| 1. I understand that any direct quotations I provide may be included anonymously in research reports, teaching, presentations and publications. | ***Radio button*** |
| 1. I understand that my data will be anonymised 12 years after the trial end date (all personally identifiable data, including consent forms will be deleted) and that the anonymised data will be entered into the UCL Data Repository and retained for at least 20 years from the trial end date. | ***Radio button*** |
| 1. I consent to participate in the APPROACH study. | ***Radio button*** |
| 1. I give consent for the APPROACH study team to access my NHS number from the hospital from which I was recruited and share my NHS number/other information about me with the national bodies that hold the National Cancer Registration and Analysis Service (NCRAS) or the linked Hospital Episode Statistics (HES) registries at the time (currently Public Health England/NHS Digital) in order to identify me in these registries. I understand that this means that information about my cancer diagnosis, hospital visits, treatment information and my health status will be shared with the APPROACH team for up to 12 years after the study has finished. If I withdraw from the study, I will be asked if I am still willing for the APPROACH team to access information about my health status from he NCRAS/HES databases, if I had previously provided permission for this. | **Radio buttons:**  **Yes and No** |

Name

Date

Signature

Telephone number

Email address

Home address

On the next page you will be shown a PDF of all the information you have read here and your responses. You will be able to download this for your records if you wish. If you would also like us to email you a copy of this PDF please click here. ***Radio button***

**NEXT**

*Page Break*

*A PDF of the information sheet and consent responses will be shown on the screen and participants can download or print this.*

*Participants then confirm they are happy with their responses and submit this form.*
